# Rab GTPases in Osteoclastic Bone Resorption and Autophagy

**DOI:** 10.3390/ijms21207655

**Published:** 2020-10-16

**Authors:** Michèle Roy, Sophie Roux

**Affiliations:** Rheumatology, Faculty of Medicine, University of Sherbrooke, Sherbrooke, QC J1H 5N4, Canada; Michele.Roy@USherbrooke.ca

**Keywords:** osteoclasts, endomembrane machinery, bone resorption, autophagy, Rab GTPases, Rab GAP

## Abstract

Small guanosine triphosphate hydrolases (GTPases) of the Rab family are involved in plasma membrane delivery, fusion events, and lysosomal and autophagic degradation pathways, thereby regulating signaling pathways and cell differentiation and function. Osteoclasts are bone-resorbing cells that maintain bone homeostasis. Polarized vesicular trafficking pathways result in the formation of the ruffled border, the osteoclast’s resorptive organelle, which also assists in transcytosis. Here, we reviewed the different roles of Rab GTPases in the endomembrane machinery of osteoclasts and in bone diseases caused by the dysfunction of these proteins, with a particular focus on autophagy and bone resorption. Understanding the molecular mechanisms underlying osteoclast-related bone disease development is critical for developing and improving therapies.

## 1. Introduction

Bone remodeling is a dynamic physiological process in which bone resorption is followed by the formation of new bone. Osteoclasts, the cells responsible for resorption, are highly polarized and mobile, indicating that they undergo intense membrane remodeling [1]. Membrane trafficking in these cells is essential not only for cell homeostasis and viability but also for bone resorption. Membrane trafficking includes processes involved in the movement of various macromolecular cargoes via membrane-bound transport vesicles and can be divided into the secretory and endocytic pathways [2]. While macroautophagy (hereinafter called autophagy) relies on secretory and endocytic pathways for the directed recycling or degradation of cargoes [3,4], transcytosis in polarized cells involves the transport of cargo from one side of the cell to the other or redistribution of the plasma membrane [5]. As small Rab guanosine triphosphate hydrolases (GTPases) are essential for the regulation of the vesicular traffic [6], they play a major role in osteoclasts. We reviewed the current knowledge regarding the network of Rab GTPases in these highly active cells with a focus on two interrelated aspects, which are osteoclastic bone resorption and autophagy, as membrane traffic is crucial for both these processes. As the available data on Rabs in osteoclasts is scarce, especially in humans, this review provides valuable insights into Rabs and their regulatory proteins as well as emerging data on Rabs in osteoclast-rich osteopetrosis, and Paget’s disease of bone.

## 2. Major Vesicular Trafficking Pathways in Osteoclasts: Bone Resorption and Autophagy

**Figure 1:**
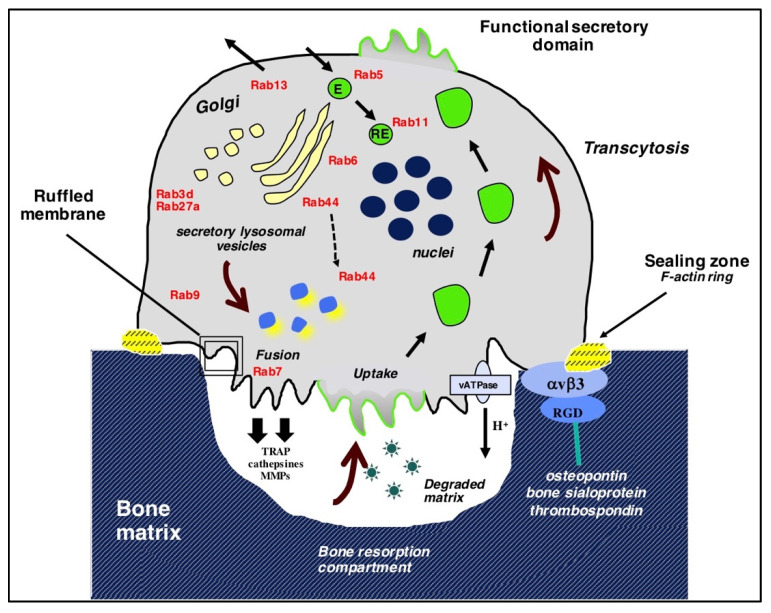
Bone resorption: a multi-step process involving vesicle trafficking. Osteoclasts polarize and reorganize their cytoskeleton upon adhering to the bone. The sealing zone (an actin-rich organelle-free zone) is formed by a peripheral belt of adhesive structures, which delimits the ruffled border, a highly specialized area consisting of membrane expansions directed toward the bone. The ruffled membrane is formed as a result of polarized vesicular trafficking and extensive fusion and plays a critical role in the degradation of bone matrix. Degraded bone matrix products are internalized at the uptake zone and transported by transcytosis toward the functional secretory domain. Several Rab proteins identified in osteoclasts have been added (in red). E: endosome; RE: recycling endosomes. Adapted from [8,13,14].

Osteoclasts are multinucleated cells derived from hematopoietic cells of the monocyte-macrophage lineage and are formed by the fusion of their mononuclear precursors [7]. They are highly mobile and alternate between the migratory and bone-resorbing stages, showing remarkable changes in their phenotype during these phases. Upon adhesion to the bone, osteoclasts become polarized and reorganize their cytoskeleton (Figure 1). A sealing zone is formed by densely packed actin-rich podosomes that delimit the ruffled border, which is a highly specialized area consisting of membrane expansions directed toward the target bone surface. The ruffled membrane is formed by the polarized trafficking of acid vesicles of the lysosomal pathway that massively fuse with the bone-facing membrane. This considerably increases the surface area of the ruffled membrane by forming highly convoluted membrane structures, and this plays a critical role in the degradation of the bone matrix via acidification by vacuolar H^+^-ATPases that are inserted in the plasma membrane and the proteases that are released in the resorption compartment during the spillage of secretory vesicles [8,9]. In non-resorptive or migrating osteoclasts, the sealing zone switches to a podosome belt, and the relaxed osteoclasts undergo depolarization [10]. In osteoclasts, the major vesicular trafficking pathways include those related to bone resorption, which depends on the formation of the ruffled membrane as a result of vesicle trafficking and fusion as well as the transcytosis of degraded products from the resorption lacuna toward the apical membrane [11,12].

**Figure 2:**
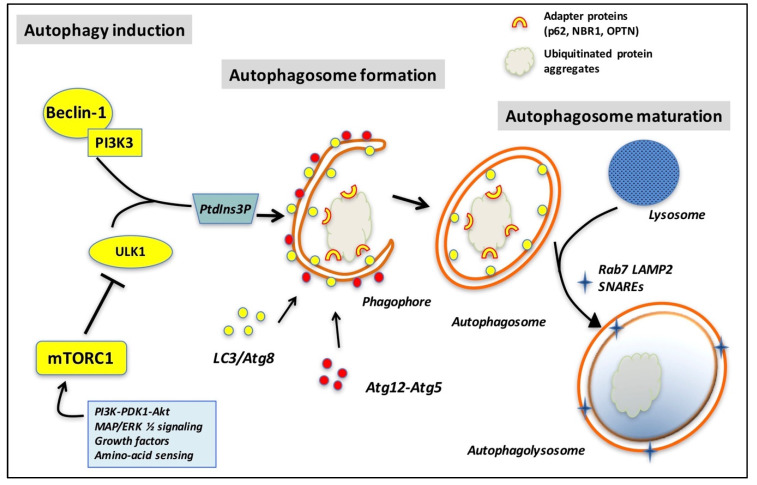
Basics of autophagy: autophagy starts at a membrane core assembly site, which requires the activity of Beclin1- phosphatidylinositol 3-kinase (PI3K) and UNC51-like kinase 1 (ULK1) complexes. Upstream signaling pathways regulate ULK1 complex activity, including PI3K/protein kinase B (PKB or Akt) and extracellular-signal regulated kinase (ERK), which function via mammalian target of rapamycin complex 1 (mTORC1), a potent inhibitor of autophagy. Vesicle expansion requires the Atg5-Atg12 conjugate and phosphatidyl inositol ethanolamine (PE)-conjugated LC3-II, both attached to the autophagosome membrane. Finally, fusion with a lysosome produces the autophagolysosome, the contents of which are degraded. Other abbreviations: Beclin1: BCL-2 interacting myosin/moesin-like coiled-coil protein 1; LC3: light chain 3 [Atg8 (yeast) is called LC3 in mammals]; PtdIns3P: phosphatidylinositol 3-phosphate; SNAREs: N-ethylmaleimide-sensitive factor attachment protein receptors; WIPI: WD repeat domain phosphoinositide-interacting protein. Adapted from [14].

Autophagy is a trafficking pathway that delivers cargo in double-membranous autophagosomes to the lysosomes for degradation and recycling (Figure 2). All the stages of formation of autophagosomes, from elongation to vesicular fusion and maturation, are dependent on the supply of membranes with the appropriate properties, thus indicating the involvement of vesicle trafficking proteins, such as Rab GTPases [15]. In osteoclasts, this pathway has been shown to be involved in oxidative stress- and hypoxia-induced differentiation [16,17]. Furthermore, the inhibition of autophagy by mammalian target of rapamycin complex 1 (mTORC1) promotes osteoclast survival and receptor activator of NF-κB ligand (RANKL)-induced formation and activation of osteoclasts [18,19]. In addition to their role in the formation of autophagosomes, some autophagy-related proteins, including LC3B, Atg5, and Rab7, are also involved in the formation of the ruffled border and sealing zone, fusion of secretory lysosomes, and subsequent bone resorption [20,21]. It has also been demonstrated that autophagy promotes osteoclast migration owing to its role in the disassembly of the podosomes via LC3-mediated degradation of kindlin-3, which is a major podosome-adaptor protein [22].

## 3. Rab GTPases and Their Regulators: Guanine Nucleotide Exchange Factors (GEFs), GTPase-Activating Proteins (GAPs), and Guanosine Diphosphate (GDP) Dissociation Inhibitors (GDIs)

Small GTPases of the Ras superfamily include the Ras, Rho, Ran, Arf, and Rab families, which are central regulators of signaling networks and cytoskeleton dynamics [23]. The functions and partners or effectors of most of the 70 identified members of the human Rab subfamily are not known [24,25]. Rab GTPases regulate different steps of vesicular transport (from budding to vesicle formation, transport, and fusion within a target membrane site) and ensure precise delivery of cargoes [26]. They function by recruiting effectors, such as sorting adaptors, cytoskeletal motor proteins, tethering factors, kinases, and phosphatases, and the spatially and temporally regulated Rab cascades contribute to the structural integrity and polarity of the secretory and endocytic pathways [6,25]. Rab GTPases predominantly integrate and mediate intracellular trafficking signals as scaffold proteins and possess multiple binding domains for regulatory molecules and downstream effectors [27]. Most Rab GTPases are ubiquitous and are grouped into subfamilies according to subcellular localization and function [27], although a single endomembrane compartment can harbor multiple Rabs in functionally distinct microdomains [25]. In addition, differential functions of some Rab GTPases have been observed in different cell types, for example, Rab13 that is involved in glucose transporter traffic in muscle cells but not in osteoclasts [28].

**Figure 3:**
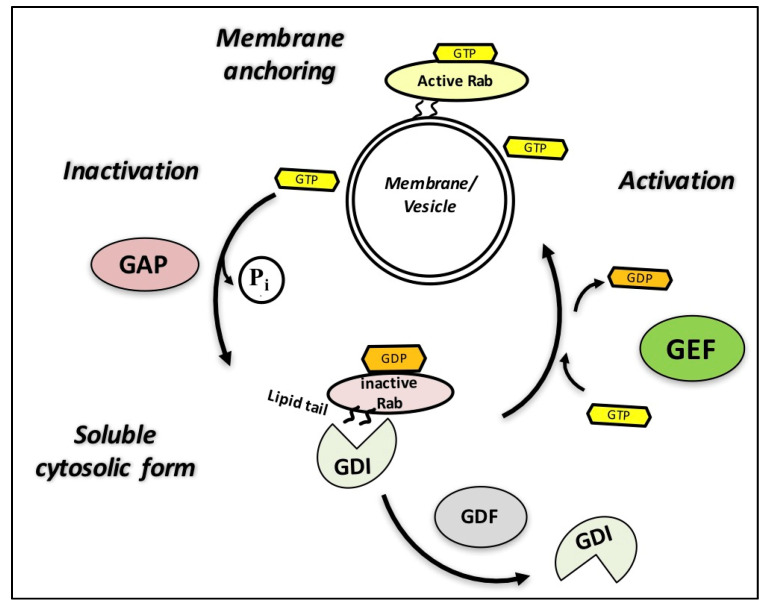
Activation/deactivation cycle of Rab guanosine triphosphate hydrolases (GTPases). In their active GTP-bound form, Rab GTPases are attached to the inner membrane and recruit various effectors until they are inactivated via hydrolysis of GTP to guanosine diphosphate (GDP) after binding to a GTPase-activating protein (GAP). GDP dissociation inhibitors (GDIs) solubilize inactive Rabs, allowing for their release into the cytosol. Membrane-associated GDI displacement factors (GDFs) mediate the delivery of Rab GTPases to new target membrane structures via GDI release. Guanine nucleotide exchange factors (GEFs) then activate Rabs by changing GDP for a GTP, thereby initiating a new cycle. Adapted from [29,31].

Rab GTPases can reversibly attach to membranes and bind GDP or GTP, switching between the active GTP-bound state, which recruits effectors, and the inactive GDP-bound state. Similar to all members of the Ras superfamily, Rab GTPases require C-terminal post-translational prenylation (lipid tail), which includes the addition of geranylgeranyl hydrophobic molecules, for associating with cytoplasmic membranes and vesicles [26]. After forming a stable complex with a Rab escort protein (REP) for facilitating their prenylation, the newly formed geranylgeranylated Rab GTPases are delivered to their target membrane. Once located at the appropriate membrane site, Rab GTPases are activated by GEFs, and the bound GDP is replaced by GTP. Active Rab GTPases can then interact with effectors via specific microdomains to perform their functions until they are inactivated by the hydrolysis of GTP to GDP after binding to a GAP. GDI solubilizes inactive prenylated Rab GTPases by binding to the lipophilic groups, thus allowing for their release into the cytosol. Membrane-associated GDI displacement factors (GDFs) mediate the delivery of Rab GTPases to new target membrane structures via the release of GDI, and the positioning of GTP is catalyzed by GEFs, which activate the Rab GTPases for their next cycle (Figure 3) [29,30].

At least six different types of Rab-activating GEFs, including those of the differentially expressed in normal and neoplastic cells (DENN), vacuolar protein sorting 9 (Vps9), and Sec2 domain families, as well as multi-subunit transport protein particle (TRAPP) complexes, with approximately 40 putative RabGEFs, have been characterized in humans [23]. Some GEFs associate with specific Rab GTPases, whereas others activate several Rab GTPases; therefore, a single Rab GTPase may be activated by different types of GEFs [32]. Unlike the low structural homology between GEFs, GAPs essentially belong to a single protein family consisting of highly conserved catalytic TBC (Tre-2/Bub2/Cdc16) domains (TBC/RabGAPs), with more than 40 different members in humans, except the TBC domain-free Rab3GAP complex that targets members of the Rab3 family. RabGAPs are less diverse than their Rab targets, and each of them may inactivate multiple Rab GTPases [29]. Interactions between GAP or GEF and a specific Rab GTPase may not regulate this Rab GDP/GTP state but may regulate that of a neighboring Rab GTPase [25,32,33].

## 4. The Rab GTPase Network in Autophagy

### 4.1. Rab GTPases Are Mainly Involved in the Early Steps of Autophagosome Formation

Rab GTPases regulate vesicular traffic events, including vesicle tethering, transport, and fusion. Several Rabs have been associated with the induction of autophagy (Rab18 and Rab37) [34,35] and regulate autophagosome biogenesis from the endoplasmic reticulum (Rab1, Rab5, and Rab32) and the Golgi apparatus (Rab33B) or facilitate the fusion of multivesicular bodies or recycling endosomes with the autophagosome (Rab11, Rab12, and Rab23) [36,37].

Rab1 and Rab5 appear to be required for autophagosome biogenesis from the endoplasmic reticulum, and the GEF TRAPPIII may activate Rab1 [36]. Rab5 has been implicated in the regulation of mTORC1 signaling, ATG5-ATG12 conjugation, and PtdIns(3)P production at the phagophore via the recruitment of type III PI3K, as well as in autophagosome closure [38]. ULK1/2, which is activated by the suppression of mTORC1 activity, mediates the phosphorylation of DENND3, a Rab12 GEF. Activated GTP-Rab12 is localized to the recycling endosomes and facilitates autophagosome trafficking [39]. Additionally, interactions between ULK1, Rab11, and TBC1D14 have been shown to transport the recycling endosomes to the phagophore assembly site during starvation-induced autophagy in HEK293 cells [40]. Similar to Rab1, Rab32 is associated with mTORC1 signaling as well as membrane trafficking from the endoplasmic reticulum, thus allowing for phagophore expansion [31]. Rab18 has been characterized as a positive regulator of early autophagy in human fibroblasts and is dependent on Rab3GAP1/2, which is a RabGEF for Rab18 [34]. Activated Rab37 has been implicated in the formation of phagophores upon the induction of autophagy via interactions with the ATG5-12/16L complex to promote the elongation of isolation membranes and expression of LC3-II [35].

Other Rab GTPases are involved in non-canonical autophagy, which represents a form of autophagy that bypasses certain canonical autophagy-related proteins. Thus, Rab9, which is involved in vesicle trafficking between the *trans*-Golgi and endosomes, has been implicated in the ATG5/ATG7/LC3-II-independent autophagy [37,41,42].

### 4.2. Late Steps of Autophagolysosome Formation

Rab GTPases are rarely involved in the later stages of autophagy, including fusion of autophagosomes with lysosomes leading to autophagolysosomes (Rab8B, Rab21, and Rab24). Upon starvation-induced autophagy, Rab21 is activated by myotubularin-related protein 13 (MTMR13), a DENN domain GEF, and contributes to the formation of autophagolysosomes via interactions with its effector Vesicle-Associated Membrane Protein 8 (VAMP8), which is an R- soluble *N*-ethylmaleimide-sensitive factor attachment receptor (SNARE) that mediates membrane fusion [43]. Rab24 has been shown to be required in the late steps of autophagy and is involved in the clearance of autophagolysosomes [44]. 

Some Rab GTPases regulate both the early and late stages (Rab7, Rab9A, and Rab33B) of autophagy. Rab7 is among the best characterized Rab GTPases involved in vesicular trafficking, and plays key roles in endosome maturation, lysosome positioning, and lysosome-related organelle biogenesis [31,45]. Rab7 is involved in different steps of the autophagy process, including the regulation of mTORC1 activity, nascent autophagosome biogenesis via pleckstrin homology domain-containing family F member 1 (PLEKHF1)/phafin1, autophagosome microtubular transport via LC3-interacting effector FYVE-coiled coil containing 1 (FYCO1), and fusion with late endosomes or lysosomes via the homotypic fusion and vacuole protein sorting (HOPS) complex and its associated SNARE and pleckstrin homology domain containing protein family member 1 (PLEKHM1) [31,46,47]. Rac1, a GTPase of the Rho family, interacts with RabGAP Armus (TBC1D2A), which is known to inactivate Rab7, indicating that this RabGAP may regulate and co-ordinate both Rac1 and Rab7 activities during autophagy [48].

### 4.3. GAPs in Autophagy

Among RabGAPs, TBC1D5, TBC1D14, and the non-TBC Rab3GAP complex are involved in the formation of autophagosomes, while TBC1D2 and TBC1D25 are associated with autophagosome-lysosome fusion [15]. TBC1D20 has also been identified as a TBC/RabGAP for Rab1 and is required for autophagosome maturation [49]. TBC1D25 binds to various Rabs, including Rab33B, which is a recently discovered binding partner of LC3 that contributes to autophagolysosome maturation [50].

## 5. Rab GTPases in Osteoclasts

Genes encoding at least 26 small GTPases are expressed in resorbing human osteoclasts; however, only Rab13 has been studied at the protein level in these cells (Table 1) [28]. Among GEFs and GAPs, only RabGEF RIN3 and RabGAP TBC1D25 are reportedly expressed in human osteoclasts [51,52], although their functions and partners remain unclear. In rodents, the expression of most Rab GTPases in osteoclasts, including Rab1, Rab2b, Rab3, Rab5, Rab6, Rab7, Rab9, Rab10, Rab11b, Rab14, Rab18, Rab27a, and Rab44, has been reported at the protein level, whereas that of Rab4, Rab35, and Rab38 has only been reported at the mRNA level [1,8,11,53,54,55,56,57,58]. Some Rab GTPases expressed by osteoclasts are involved in autophagy, as described earlier, whereas others appear to be involved in bone resorption (Rab3d, Rab27a, and Rab44) or both autophagy and resorption (Rab5c, Rab7, and Rab11b) (Table 1).

### 5.1. Rab7 Is Crucial for Osteoclast Bone Resorbing Activity 

Rab7 trafficking events are best described in osteoclasts, and strong evidence indicates a major role of this Rab GTPase in membrane trafficking and osteoclast activities. In rodent osteoclasts, Rab7 is highly expressed and is mainly localized to the ruffled membrane of resorbing cells, while displaying a perinuclear distribution typical of late endosomes in inactive osteoclasts [59]. The downregulation of Rab7 in osteoclasts has been shown to impair the formation of F-actin rings, inhibiting cell polarization and reducing bone resorption, which suggests its role in vesicular trafficking during bone resorption [60]. Via direct interactions with Rab7, the α3 subunit of the V-ATPase can act as Rab7-GEF allowing its recruitment to the membrane of secretory lysosomes [61]. The accessory subunit (ATP6AP1) of V-ATPase, which is involved in the regulation of lysosomal trafficking to the ruffled membrane, may also act via interactions with Rab7 [62].

PLEKHM1, a multivalent adaptor protein in the endomembrane system [63] and a specific effector for the terminal fusion of autophagosomes and lysosomes [47], has been shown to colocalize with GTP-bound Rab7 in late endosomes/lysosomes. PLEKHM1 can act as a Rab7 effector for osteoclast vesicle trafficking via molecular complexes involving DEF8 (differentially expressed in factor dependent cell-Paterson (FDCP) myeloid progenitor cells 8) [64] or TRAFD1 (TRAF-type zinc finger domain containing 1) [65]. Furthermore, osteoclasts from *PLEKHM1*-deficient mice or those from patients with loss-of-function mutations in *PLEKHM1* have compromised resorption activity due to the absence of ruffled membranes [64,66].

Rac1, a GTPase of the Rho family, has been shown to interact with Rab7 at the fusion zone of the ruffled membrane in the resorbing osteoclasts of rodents, suggesting its role in the microtubular transport of lysosomes to the bone-facing plasma membrane for forming the ruffled expansion [67].

### 5.2. Other Rab GTPases Involved in Osteoclast Activities

In human osteoclasts, *RAB13* is highly upregulated during osteoclast differentiation, although it is not involved in bone resorption, transcytosis, endocytosis, and glucose transport. The downregulation of Rab13 does not affect osteoclast differentiation, and in mature osteoclasts, Rab13 is localized to small vesicular structures between the *trans*-Golgi networks and basolateral membranes, suggesting associations with the osteoclast secretory functions [28], possibly through interacting with endospanin-2, a small transmembrane protein [68]. 

The expression of Rab3 isoforms with known roles in exocytosis has been previously investigated in murine osteoclast precursors, which expressed Rab3a and Rab3b/c [69]. In further studies, Rab3d has been reported as the major osteoclastic Rab3 isoform, and Rab3d knockout mice were found to have high bone mass and impaired osteoclastic bone resorption. Furthermore, osteoclasts from Rab3d-deficient mice displayed the formation of normal F-actin ring and podosomes, but abnormal ruffled borders. In osteoclasts, Rab3d has been associated with a non-lysosomal post-Golgi trafficking step required for bone resorption [70]. Tctex-1, a light chain of the cytoplasmic dynein microtubule motor complex, has been identified as an Rab3d partner in the transport of secretory vesicles to the ruffled membrane during bone resorption [71].

Rab5c has been associated with early endosomes, and Rab11b, one of the most abundant Rab GTPases in rodent osteoclasts, is localized to perinuclear recycling compartments. Although neither Rab5c nor Rab11b have been detected at the ruffled membrane, they have been demonstrated to contribute to upstream events involving resorption-related vesicular transport [1]. Rab6 is localized to the Golgi apparatus in osteoclasts, although their roles in these cells remain unclear [26].

Rab9 is involved in late endosome trafficking to the *trans-*Golgi network in other cell types [26,57]. Owing to its colocalization with Rab7 in the late endosomes around the nuclei, Rab9 is believed to be involved in this trafficking pathway in osteoclasts. Furthermore, a fraction of Rab9 localizes to the plasma membrane adjacent to the bone matrix in a pattern complementary to that of Rab7 in active osteoclasts, although its function has not been further investigated [1].

In mice, the expression of Rab27a mRNA increased, whereas that of Rab27b decreased during osteoclast differentiation. Osteoclasts from Rab27a-deficient mice showed defects in the formation of actin rings, abnormal subcellular localization of lysosome-associated membrane protein (LAMP2) and cathepsin K, and impaired bone resorption, suggesting a role of Rab27a in the transport of secretory lysosomes in these cells [56]. In addition, Rab27a might also favor osteoclast formation, as the inhibition of its expression by miR-124 was associated with the inhibition of osteoclastogenesis [72].

Although RANKL strongly induces the expression of Rab38 in murine osteoclasts, Rab38 may be dispensable for the formation or function of osteoclasts [53]. Finally, Rab44 is a GTPase highly expressed in undifferentiated hematopoietic cells [73]. Rab44 was detected in murine osteoclasts in the Golgi apparatus and lysosomes and was shown to inhibit osteoclast differentiation by modulating intracellular Ca^2+^ levels [58].

Transcytosis is a vesicular trafficking pathway that has been observed in resorbing osteoclasts and involves endocytosis of degraded products at uptake zones of ruffled membranes and vesicle transport to the functional secretory domain for exocytosis. Some Rab GTPases play major roles in transcytosis in polarized cells, driving vesicular traffic from basolateral to apical membranes, as described for Rab17 in epithelial cells [55]; however, until now, no Rab GTPase has been associated with osteoclast transcytosis.

## 6. Rab GTPases in Human Bone Diseases

Unlike the direct involvement of Rabs reported to be upregulated in some cancers or whose genes have been found mutated in neurological disorders [74], only indirect evidence implicating individual Rabs has been provided in bone diseases, relying on the aberrant expression or mutations of their interacting proteins: effectors (PLEKHM1 as Rab7 effector) or regulatory proteins (TBC1D25, RIN3).

### 6.1. Osteopetrosis

Osteopetrosis is an inherited heterogeneous bone disease characterized by the inability to resorb bone, with consequential high bone mass and generalized osteosclerosis [75]. In humans, autosomal recessive osteopetrosis (ARO) is mainly (in >70% cases) associated with mutations in two genes: *TCIRG1,* encoding the α3 subunit of v-ATPase [76], and *CLCN7*, encoding anion transporter CLC-7 that works with v-ATPase to acidify the bone while maintaining electroneutrality [77].

Mutations in *OSTM1*, *SNX10,* and *PLEKHM1* are rare and are also associated with forms of osteopetrosis characterized by the presence of non-functional osteoclasts (osteoclast-rich osteopetrosis). *OSTM1* encodes osteopetrosis-associated transmembrane protein 1 (OSTM1), a β-subunit of CLC-7 [78], also involved in osteoclast membrane trafficking [79]. *SNX10* encodes a sorting nexin (nexin 10) involved in lipid attachment and cargo sorting in the endosomal pathway [80,81]. In the presence of a *SNX10* mutation, osteoclasts exhibit defective ruffled membranes and are unable to resorb bones [80]. Loss-of-function mutations in *PLEKHM1* result in an intermediate or severe form of osteopetrosis in humans, with no or underdeveloped ruffled membranes in patient-derived osteoclasts [66], or altered endocytosis and autophagy in cells expressing the mutant gene [82]. These mutations reflect altered interactions of PLEKHM1 with Rab7 [66,82], leading to defective endosomal/lysosomal vesicle transport and impaired bone resorption [64].

Osteopetroses with developmental defects of osteoclasts (osteoclast-poor osteopetrosis) are more rare, secondary to diseases caused by mutations in *TNFRSF11A* or *TNFSF11,* encoding RANK and RANKL, respectively [83].

### 6.2. Paget’s Disease of Bone (PDB)

PDB is characterized by a focal and disorganized increase in bone turnover. As the initial phase of PDB involves excessive bone resorption, impaired osteoclasts are considered the primary cellular consequence of PDB [84]. Pagetic osteoclasts are larger and more numerous than normal osteoclasts; they are overactive and hypersensitive to osteoclastogenic factors and are resistant to apoptosis [85]. As inclusion bodies in pagetic osteoclasts resemble the sequestosome-1 or SQSTM1/p62 aggregates observed in diseases involving defective autophagy, the pathogenesis of PDB possibly involves the impairment of autophagy [86]. In previous studies, defects in autophagy flux were observed in PBD osteoclasts or Cos-1 cells harboring a PDB-associated p62 mutation, suggesting accumulation of non-degradative autophagosomes [87,88]. The activation of TBK1 (TANK binding kinase) and TBK1-induced IL-6 production may also contribute to the generation of PDB osteoclasts [89]. Rab8B has been shown to recruit TBK1 to autophagic organelles and contribute to autophagy-mediated antimicrobial defenses, such as the autophagic elimination of *Mycobacterium tuberculosis* via the phosphorylation and activation of p62 [31,90].

In a previous study, we identified alternative RNA splicing events in *TBC1D25*, which encodes TBC1 domain family member 25 (TBC1D25) and demonstrated that TBC1D25 was expressed in human osteoclasts [51]. In particular, during analyses of the two spliced isoforms of *TBC1D25,* we observed a slight but significant decrease in mRNA and protein expression of the long isoform in pagetic osteoclasts compared to that in the healthy osteoclasts; these observations were independent of mutations in the gene encoding SQSTM1/p62 associated with PDB [51]. Residues 134-136, which interact with LC3 as well as the TBC (Rab-GAP) domain, are missing in the short isoform, suggesting that alternative splicing regulates a proportion of active TBC1D25. Among the known osteoclast-expressed Rab GTPases, Rab13, Rab33B, and Rab34 may interact with TBC1D25 [50,91]. Finally, RIN3 is a GEF for the small GTPases, Rab5 and Rab31, and has been associated with endocytosis, vesicular trafficking, and signal transduction. Although the specific role of RIN3 in bone metabolism has not been studied, genetic variants of *RIN3* have been reported to predispose to PDB [52].

## 7. Rab GTPases as Therapeutic Targets 

Small GTPases are crucial signaling proteins that regulate various processes necessary for osteoclast function, such as cytoskeletal organization, vesicular trafficking, and cell survival. Post-translational prenylation is essential for the membrane-targeting and function of small GTPases, and disrupted prenylation may result in osteoclast apoptosis [92]. Bisphosphonates are anti-catabolic drugs that directly suppress osteoclast activity and induce osteoclast apoptosis and are widely used to treat bone disorders characterized by increased bone resorption, such as PDB, osteoporosis, and malignant osteolysis. The bone specificity of bisphosphonates (BPs) lies in their strong affinity for hydroxyapatite, and osteoclasts are subsequently mainly exposed to BPs when internalising these molecules during bone resorption. Small GTPases, such as Ras, Rho, and Rab, are targets for nitrogen-containing bisphosphonates (N-BPs) that inhibit their post-translational prenylation [93]. N-BPs inhibit farnesyl pyrophosphate (FPP) synthase of the mevalonate pathway, thus depleting cells of FPP and geranylgeranyl pyrophosphate, isoprenoid lipids both required for the prenylation of small GTPases [94]. A defect in the prenylation of small GTPases prevents their membrane localization and leads to their cytosolic accumulation [94]. As a result, the mislocation of small GTPases, dysregulation of cytoskeletal rearrangements, and the disruption of the ruffled membrane formation during osteoclast polarization have been observed after treatments with N-BPs [11,95]. A defect in protein prenylation may also activate signaling pathways, as the ability of regulatory proteins such as GAPs and GDIs to modulate small GTPase activity also depends on prenylation [94]. 

Although N-BPs are more likely to affect geranylgeranyl small G-proteins [11], they do not specifically inhibit the prenylation of Rab proteins. Phosphonocarboxylate analogs of N-BPs do not inhibit FPP synthetase, but they do inhibit the Rab geranylgeranyltransferase (RGGT), specifically inhibiting Rab protein prenylation. A phosphonocarboxylate analog of risedronate inhibited bone resorption in vitro [96]. Similarly, although osteoclasts from *gunmetal* mice have reduced RGGT activity, they are formed normally and polarize into sealing zones without any disruption of F-actin rings. These osteoclasts exhibit reduced bone resorption activity because of impaired ruffled border formation in vitro. However, the remaining prenylated Rab proteins were sufficient for maintaining normal bone resorption in vivo. Furthermore, Rab7 remained 86% prenylated in these mice, and not all Rab proteins were affected by the reduction in RGGT activity [57].

Although prenylation is essential for the localization of Rab GTPases to specific intracellular compartments, targeting of prenylation may not be sufficient to alter its functions in osteoclasts. Indeed, while mice deficient in Rab3D develop osteopetrosis with reduced osteoclast activity and irregular ruffled membranes, disruption of osteoclast bone resorption was dependent on guanine nucleotide binding (formation of GTP-bound Rab3D) and not on RAb3D prenylation status [70].

## 8. Conclusions

Small GTPases of the Rab family play major roles in osteoclast function, particularly in autophagy and bone resorption via ruffled membranes. The mechanisms of lysosome trafficking and autophagy involving Rab GTPases in osteoclasts may be unique to this secreting cell type, and further studies on Rab GTPase activities and their regulators in osteoclasts are required. Indeed, while Rab proteins play major roles in cell homeostasis and numerous Rabs have been now identified, the functions of most of them remain unknown, as well as their effectors and regulators, particularly in osteoclasts. Currently, few effectors have been described in osteoclasts such as Rab7 effectors (Rac1, PLEKHM1), or Rab3d effector (Tctex-1), and only one RabGEF (RIN3), and one TBC/RabGAP (TBC1D25) have been reported to be expressed in human osteoclasts. In addition, the determinants of tissue- and cell-type specificity of these small GTPases remain to be identified [97]. Thus, the identification of the different Rabs involved in each step of secretory vesicle trafficking and their regulators (GEFs, GAPs) in osteoclasts, particularly in humans, would improve our knowledge on osteoclast biology and bone diseases, enable identification of specific therapeutic targets, and facilitate the study of bone resorption using these potential biomarkers.

## Figures and Tables

**Table 1:** Identification of Rab GTPases in osteoclasts and their involvement in autophagy and bone resorption.

Rab GTPases	Human Osteoclasts	Rodent Osteoclasts		Autophagy *(*Non-Osteoclastic Cells*)*	Bone Resorption
	Gene Expression	Gene Expression	Protein		
1		X (b)	X	X	
2			X (b)		
3 (a–d)	X (c)		X (a, b/c, d)		X (d)
4 (a, b)	X (a)	X (b)			
5	X (a, b)	X (c)	X	X	X (c)
6			X		
7	X	X	X	X	X
8	X (b)			X	
9 (a, b)	X (a)	X	X		
10	X		X		
11 (a, b)	X (a)	X (b)	X (b)	X	X (b)
12	X			X	
13	X				--
14	X		X		
15, 17, 19	--				
18	X		X	X	
20	X				
21	X			X	
22a	X				
23	X			X	
24	X			X	
25, 26	--				
27 (a, b)	X (a)	X (a)	X (a)		X (a)
28, 29	--				
30	X				
31	X				
32	X			X	
33 (a, b)	X (a, b)			X (b)	
34	X				
35		X			
36, 37	--			X	
38	X	X			--
39a/b, 40	--				
44		X	X		X ^1^

^1^ indirect impact (osteoclast formation). [X detected; -- not detected; Isoforms identified with lowercase letters are specified where appropriate].

## Abbreviations

Beclin 1	BCL-2 interacting myosin/moesin-like coiled-coil protein 1
DENN	differentially expressed in normal and neoplastic cells
FPP	farnesyl pyrophosphate
FYCO1	FYVE-coiled coil containing 1 (protein)
GAP	GTPase-activating protein
GDI	GDP dissociation inhibitor
GDF	GDI displacement factor
GDP/GTP	guanosine diphosphate (GDP)/guanosine triphosphate (GTP)
GEF	guanine–nucleotide exchange factor
GGPP	geranylgeranyl pyrophosphate (GGPP)
HOPS	homotypic fusion and protein sorting complex
LAMP2	lysosome-associated membrane protein 2
LC3	light chain 3 [Atg8 (yeast) is called LC3 in mammals]
MCSF	macrophage colony-stimulating factor
mTORC1	mammalian target of rapamycin complex 1
PDB	Paget’s disease of bone
Phafin1	a FYVE and PH (Pleckstrin homology) domain containing protein
PLEKHF1	pleckstrin homology domain-containing family F member 1 (a.k.a. Phafin1)
PLEKHM1	pleckstrin homology domain containing, family M (with RUN domain) member 1
PtdIns3P	phosphatidylinositol 3-phosphate
RANKL	receptor activator of NF-κB ligand
REP	Rab escort protein
RIN3	Ras and Rab Interactor 3
RGGT	Rab Geranyl-Geranyl transferase
SNAREs	N-ethylmaleimide-sensitive factor attachment protein receptors
TBC	Tre-2/Bub2/Cdc16
TBK1	TANK Binding Kinase1
TRAPP	Transport protein particle
ULK1	UNC51-like kinase 1
